# Outcomes for Patients With Chronic Limb-Threatening Ischemia After Direct and Indirect Endovascular and Surgical Revascularization: A Meta-Analysis and Systematic Review

**DOI:** 10.1177/15266028241248524

**Published:** 2024-04-30

**Authors:** Arthur Tarricone, Allen Gee, Karla de la Mata, Lee Rogers, Jose Wiley, Lawrence A. Lavery, Prakash Krishnan

**Affiliations:** 1 Department of Plastic Surgery, The University of Texas Southwestern Medical Center, Dallas, TX, USA; 2 Nova Southeastern University Dr. Kiran C. Patel College of Osteopathic Medicine, Davie, FL, USA; 3 Department of Podiatry, Paley Orthopedic & Spine Institute, West Palm Beach, FL, USA; 4 Department of Orthopaedics, The University of Texas Health Science Center, San Antonio, TX, USA; 5 Section of Cardiology, Department of Medicine, Tulane University School of Medicine, New Orleans, LA, USA; 6 The Zena and Michael A. Wiener Cardiovascular Institute and the Marie-Josée and Henry R. Kravis Center for Cardiovascular Health, Division of Cardiology, Department of Medicine, Icahn School of Medicine at Mount Sinai, New York, NY, USA

**Keywords:** infection, diabetes, ulcer, osteomyelitis, amputation, peripheral vascular disease, peripheral arterial disease, chronic limb threatening ischemia, revascularization

## Abstract

**Purpose:**

The purpose of this review and meta-analysis is to determine the clinical outcome differences between patients with chronic limb-threatening ischemia who underwent direct versus indirect angiosome revascularization using either the surgical or endovascular approach.

**Materials and Methods:**

The data sources used for article selection included PubMed, Embase/Medline, Cochrane reviews, and Web of Science (All studies were in English and included up to September 2023). All articles included were comparative in design, including retrospective, prospective, and randomized controlled trials that compared the clinical outcomes between direct and indirect angiosome-guided revascularization in chronic limb-threatening ischemia. A random-effects model was used to determine the measure of association between direct revascularization and amputation-free survival, wound healing, and overall survival. Publication bias was assessed with both Begg’s and Egger’s test, and heterogeneity was calculated using an I^2^.

**Results:**

Data from 9 articles were analyzed and reported in this review. Direct revascularization was associated with improved amputation-free survival (odds ratio [OR]=2.632, confidence interval [CI]: 1.625, 4.265), binary wound healing (OR=2.262, CI: 1.518, 3.372), and overall survival (OR=1.757, CI: 1.176, 2.625). Time until wound healed was not associated with either direct or indirect revascularization (Standard Mean Difference [SMD]=−2.15, p=0.11). There was a low risk of bias across all studies according to the RoB 2.0 tool.

**Conclusion:**

Direct revascularization is associated with improved amputation-free survival, overall survival, and wound healing in chronic limb-threatening ischemic patients compared to the indirect approach.

**Clinical Impact:**

Preservation of the lower extremity is critical for preventing mortality and maintaining independence. The benefit of angiosome-guided revascularization for chronic limb-threatening ischemia remains controversial. The authors of this article aim to review the current literature and compare direct and indirect angiosome-guided intervention for preserving the lower extremity. Current findings suggest direct angiosome-guided intervention reduces amputation rates and improves survival; however, many trials neglect to address the multifactorial approach needed in wound care management.

## Introduction

Chronic limb-threatening ischemia (CLTI) is the most advanced stage of peripheral artery disease (PAD) and accounts for 10% of PAD patients worldwide.^1,2^ Management of CLTI requires significant clinical resources and has been estimated to cost the United States healthcare system over $12 billion per year.^1^ Chronic limb-threatening ischemia is a strong predictor for both above-the-ankle amputation and mortality.^3–5^ Preventing lower-extremity amputations (LEAs) is critical as it prolongs life and independence. LEA carries a higher risk of 5-year mortality than myocardial infarctions (MI) and cancer.^6^ Another consequence of amputation is permanent disability, where an estimated 55% of amputation patients never return to ambulatory status.^7^ Psychosocially, patients with diabetic foot pathology fear major LEA more than death.^8^

The mainstay of CLTI management is the promotion of wound healing through aggressive endovascular or surgical interventions that restore blood flow and limb perfusion.^1,9,10^ Chronic limb-threatening ischemia is a complex disease process often including a multi-level and multi-tibial disease. The foot itself is divided into 6 anatomical tissue regions known as angiosomes.^11^ Three angiosomes are supplied by the posterior tibial artery (PTA), one is supplied by the anterior tibial artery, and two are supplied by the peroneal artery (PA).^11^ Direct revascularization relies on supplying perfusion to the artery that directly supplies the area of the wound. Indirect revascularization differs by relying on interangiosomal connections known as choke vessels that are found throughout the leg and foot. This study aims to analyze the difference in outcomes for patients with CLTI who receive direct or indirect revascularization.

## Materials and Methods

A literature search was performed across 4 databases: Pubmed, Embase/Medline, Web of Science, and Cochrane Central Register of Controlled Trials. Medical Subject (MeSH) and Boolean operators were employed in the search strategy, and the final search was conducted on 17 September 2023. This systematic review and meta-analysis was conducted in accordance with the Preferred Reporting Items for Systematic Reviews and Meta-Analyses (PRISMA) Checklist. The search strategy has been registered with Research Registry under the protocol number 1735.

After all searches were populated, duplicates were removed. The remaining citations were then screened by their titles and abstracts against the inclusion and exclusion criteria to determine relevancy to the study topic. Included studies contained patients with nonhealing, lower-extremity wounds undergoing direct or indirect revascularization. Excluded studies included case reports, basic science research, non-human populations, non-English translatable studies, systematic reviews, meta-analyses, patient populations without CLTI, or absence of mention of angiosomes in the literature. The screening process was conducted by two independent reviewers (A.G. and A.T.), with any disagreements resolved through a third reviewer consensus (P.K.). A further reference list was also created to capture articles that were not found during the initial database searches but were relevant to this study.

The resulting articles were then reviewed in their full length to determine the study outcomes and extractable data. The primary outcomes that were examined in this study were amputation-free survival (AFS) defined as freedom from major amputation or death, survival defined as absence of death within the study period, and 6-month wound healing defined as re-epithelialization of the tissue defect without subsequent amputation or recurrence by 3 months. Time to wound healing was defined as the average number of days until binary wound closure.

Statistical analysis divided patients into two groups: direct revascularization and indirect revascularization. All tests for this study report findings that compare these two groups. STATA BE17 was used to compute odds ratios (OR), publication bias, and Egger’s plots. Heterogeneity was calculated using an I^2^ test. Comprehensive meta-analysis was used to generate forest plots and compute effect sizes. All meta-analyses were conducted using a random-effects model. Continuous variables were reported as means±standard deviation, and categorical variables were reported as frequency (%). A p-value of ≤0.05 was considered significant for this study.

## Results

**Figure 1.**
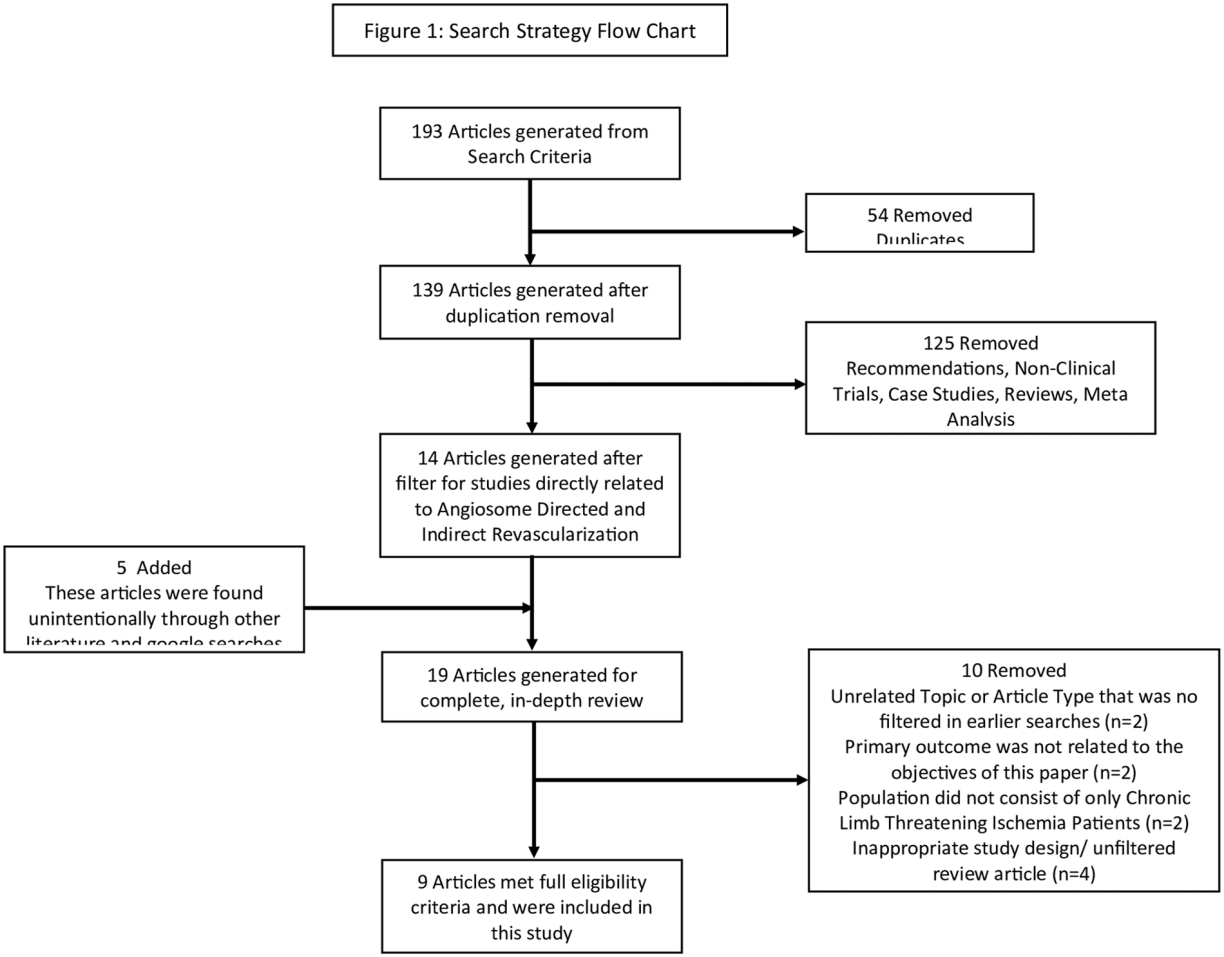
Flow chart of acquiring articles for review.

A total of 193 articles were retrieved from the initial search. After titles and abstracts were screened, 19 articles remained for full review. A total of 9 articles were included in this review and meta-analysis: 7 retrospective studies and 2 prospective studies. Risk of bias was examined through the use of the Cochrane Risk of Bias Tool 2 and was reported as low in this review (Supplemental Figure 1). Publication bias was assessed using the Egger test. There was no evidence of publication bias based on the Egger’s test (p=0.21; Supplemental Figure 2). A further breakdown of the search strategy and detailed explanations for article removal can be viewed in Figure 1.

A total of 985 patients were included across the 9 studies. The complete patient demographics, intervention modalities, and postoperative wound care techniques are presented in Table 1. Patient ages ranged between 48 and 90 years, and 313 women made up the total population. There were 542 patients who received angiosome-directed revascularization and 407 patients who received indirect revascularization. There were 725 (73.6%) patients with diabetes and 513 (52.1%) patients with ischemic heart disease (IHD). Mean follow-up period for the studies ranged from 6 months to 79 months. Five studies used the Rutherford System to identify CLTI patients, one study based the presentation of CLTI off the Trans-Atlantic Inter-Society Consensus (TASC) classification, and three studies presented only patients with ischemic ulcers and made no mention of a classification system. Three studies included patients with tissue loss, Rutherford ≥5,^12–14^ and two studies included patients with rest pain, Rutherford=4,^15,16^ in their patient population. All patients with Rutherford 1 to 3 were excluded. Ulcer grading based on the Wagner system was performed in 2 studies.^12,16^ Wound infection was determined in 2 studies: One utilized the Wound, Ischemia, Foot Infection (WIFI) score system, and one utilized the Armstrong scoring system.^13,17^ One study reported perceivable peripheral neuropathy.^12^ One study reported continuous diabetic control with average laboratory values of HbA1C.^18^

**Table 1.** Study Demographics, Intervention Methods, and Postintervention Wound Care.

Author	Total patients	Neuropathy	Iintervention method	Technology/device used	Postintervention	Artery treated
Neville et al. (2009)	Total n=56 Women=26 Diabetes=49 IHD=16	Not defined	Bypass	Saphenous vein and polytetrafluoroethylene grafts	Hyperbaric oxygen Topical growth factors (n=“one third of patients”) Split Skin Thickness Grafting (n=10) Local Flaps (n=21)	Anterior tibial (n=22) Posterior tibial (n=17) Peroneal (n=13)
Varela et al. (2010)	Total n=76 Women=65 Diabetes=61 IHD=22	Not defined	Bypass & endovascular	Stents, percutaneous transluminal angioplasty, saphenous vein grafts	Vacuum-assisted devices (n=8) Autologous platelet aggregates (n=4)	Anterior tibial (n=47) Posterior tibial (n=7) Peroneal (n=22)
Kabra et al. (2010)	Total n=64 Women=11 Diabetes=52 IHD=20	Not defined	Bypass & endovascular	Balloon angioplasty	Off-loading was advised to all patients (n=unknown) Hyperbaric oxygen (n=1) Split skin thickness grafting (n=1)	Anterior tibial (n=27) Posterior tibial (n=22) Peroneal (n=15)
Rashid et al. (2012)	Total n=154 Women=39 Diabetes=125 IHD=72	Not defined	Bypass	Saphenous vein and polytetrafluoroethylene grafts	Topical wound care (n=154) Split skin grafting (n=18) Vacuum-assisted closure (n=unknown)	Anterior tibial (n=14) Posterior tibial (n=122) Peroneal (n=5)
Lejay et al. (2013)	Total n=58 Women=11 Diabetes=58 IHD=31	Not defined	Bypass	Saphenous vein grafts	Duplex scanning for all patients at 3 and 6 months	Anterior tibial (n=48) Posterior tibial (n=6) Peroneal (n=4)
Ricco et al. (2015)	Total n=120 Women=33 Diabetes=69 IHD=66	Not defined	Bypass	Saphenous vein and polytetrafluoroethylene grafts	Wound dressing changes at each follow-up visit	Peroneal (n=120)
Alexandrescu et al. (2019)	Total n=167 Women=30 Diabetes=33 IHD=72	Semmes-Weinstein monofilament test (n=unknown)	Endovascular	Nitinol stents	Off-loading was used if necessary (n=unknown) Negative pressure wound therapy (n=124)	Not defined
Bekeny et al. (2021)	Total n=105 Women=24 Diabetes=94 IHD=27	n=54 No definition for neuropathy	Endovascular	Balloon angioplasty	Scheduled follow-up visits for dressing changes (n=unknown)	Anterior tibial (n=31) Posterior tibial (n=17) Peroneal (n=9)
Azuma et al. (2011)	Total=228 Women=63 Diabetes=184 IHD=122	Not defined	Bypass	Saphenous vein, femoral vein, arm vein, and short saphenous vein grafts	Negative pressure wound therapy (n=unknown) Fiblast—fibroblast graft factor—spray (n=unknown)	Not defined

Study publication dates ranged from 2009 to 2021. All patient demographics, intervention method, adjunctive devices, postintervention care, and the treated artery are reported in this table.

Five studies reported the use of bypass as the primary approach for revascularization,^13,14,16,17,19^ 2 studies reported the use of an endovascular approach,^12,18^ and 2 studies examined both forms of revascularization.^15,20^ There were 332/985 (33.7%) patients treated using the endovascular approach, while 650/985 (66.0%) patients were treated with bypass. Three patients were treated with hybrid techniques. Endovascular intervention differed across studies and included deployment of stents and plain old balloon angioplasty (POBA). No studies included the use of drug-eluting technology. Preoperative angiography and duplex ultrasound were performed in all studies. Four studies provided designated follow-up time points.^14,15,17,19^ Ankle brachial indexes (ABIs) were calculated in 4 studies.^12,13,15,20^ Three studies that investigated surgical revascularization identified the vessels used for proximal and distal anastomoses. Vessels for the proximal anastomoses differed across studies and included the common femoral artery, superficial femoral artery, deep femoral artery, iliac artery, PTA, and the popliteal artery. Distal anastomoses included the PA, tibio-peroneal trunk, PTA, dorsalis pedis artery, plantar artery, and peri-malleolar region. Bypass graft differed across studies; all patients in the study by Lejay et al received bypass grafts from the great saphenous vein; Kabra et al and Neville et al utilized a mixture of synthetic and autologous grafts; Ricco et al used a polytetrafluoroethylene graft if an autologous graft was not available; Azuma et al used a variety of grafts, with the great saphenous vein as the most common modality.^13–15,17,19^

Amputation-free survival was described in all studies, and overall cohort survival was described in 8/9 studies. Postintervention advanced wound closure techniques differed across studies and included skin grafts, negative pressure wound therapy, and hyperbaric oxygen therapy. Serial debridement use was mentioned in a singular study.^19^ Rashid et al^16^ reported the use of vacuum-assisted wound closure when the situation was deemed necessary, and split skin thickness grafting if a wound measured >7 cm. Kabra et al^15^ reports the use of hyperbaric oxygen, split skin grafting, and the application of topical growth factors when tissue re-epithelialization was insufficient.

**Figure 2.**
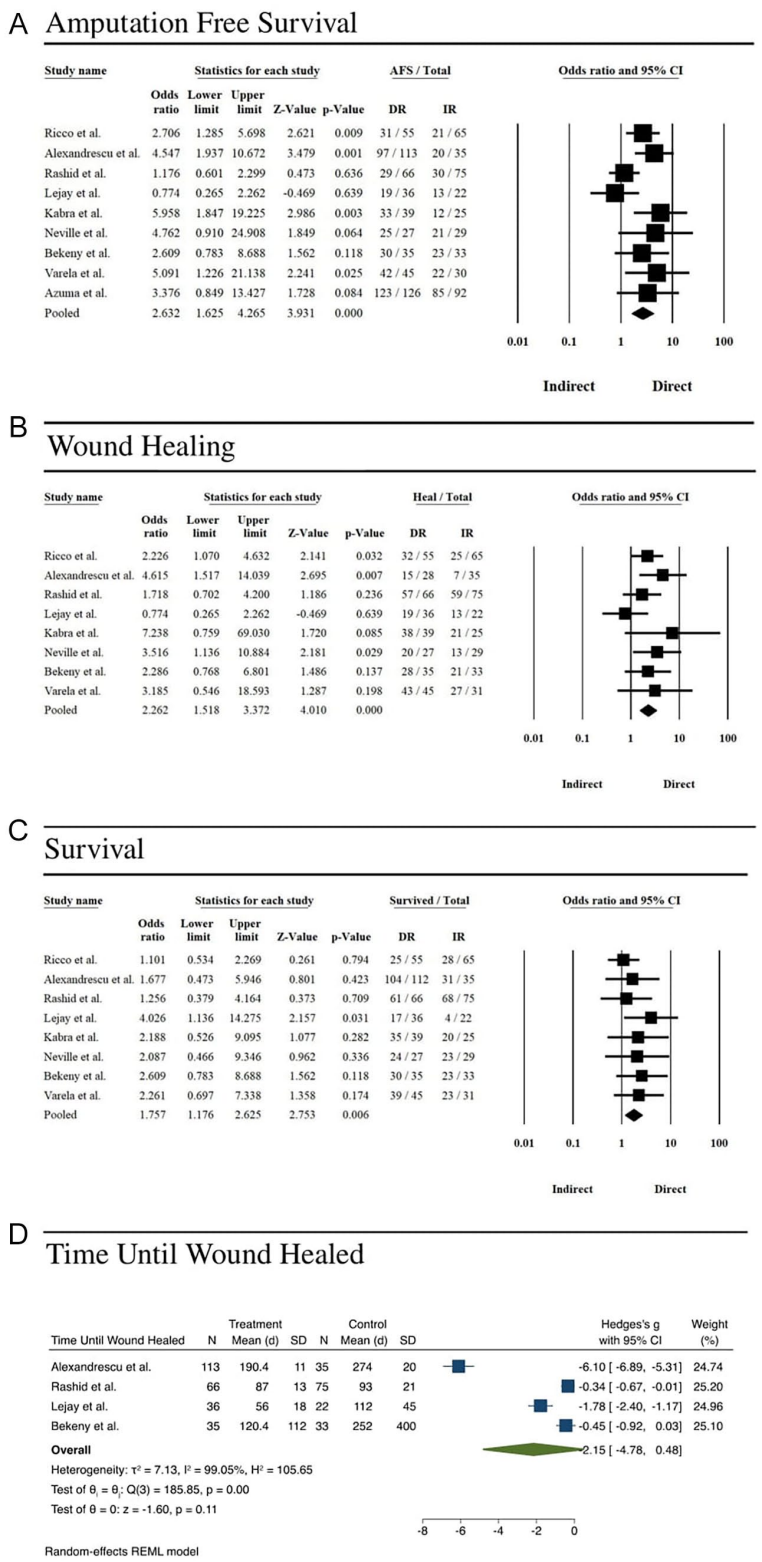
(A) Forest plot of amputation-free survival and direct revascularization. The central vertical line indicates an odds ratio of 1.0, and the black diamond indicates the average odds ratio across 9 studies. Individual study odds ratios are indicated by the black boxes, and the confidence intervals are indicated by the black horizontal lines. (B) Forest plot of wound healing and direct revascularization. The central vertical line indicates an odds ratio of 1.0, and the black diamond indicates the average odds ratio across 9 studies. Individual study odds ratios are indicated by the black boxes, and the confidence intervals are indicated by the black horizontal lines. (C) Forest plot of survival and direct revascularization. The central vertical line indicates an odds ratio of 1.0, and the black diamond indicates the average odds ratio across 9 studies. Individual study odds ratios are indicated by the black boxes, and the confidence intervals are indicated by the black horizontal lines. Indirect = indirect revascularization, Direct = direct revascularization. (D) Forest plot of time until wound healed and direct revascularization. The green diamond indicates the average standard mean difference of time until wound healed across the specified studies. Individual study standard mean differences are indicated by the blue boxes, and the confidence intervals are indicated by the blue horizontal lines.

There was an overall significant association between direct revascularization and AFS (OR=2.6, CI: 1.6–4.3), wound healing (OR=2.3, CI: 1.5–3.4), and survival (OR=1.7, CI: 1.2–2.6; Figure 2A–C). No association was observed between either forms of revascularization with time until wound healed (Figure 2D). The direct revascularization outcomes report a cumulative AFS of 429/542 (79.2%), end-of-study survival of 335/416 (80.5%), and binary wound healing 247/407 (60.7%). The average days until wound healing for patients treated with direct revascularization was 123.24 days. Indirect revascularization resulted in AFS of 247/406 cases, end-of-study survival of 220/315, and binary wound healing of 186/315. The average days until wound healing for patients treated with indirect revascularization was 178.16 days.

## Discussion

Cumulative effects of direct revascularization exhibit improved AFS, binary wound healing, and reduced mortality rates compared to indirect revascularization. However, the choice of direct versus indirect intervention did not significantly impact the time to wound healed across the collected studies. Often direct or indirect revascularization is not a choice. It is dictated by the available targets and not the ideal targets. Admittedly, this meta-analysis attempts to take a set of complex surgical decisions and dichotomize the outcomes into direct and indirect revascularization options. Wound healing and limb preservation involve many different treatments in addition to revascularization options. However, revascularization may be the single most important intervention.

Re-establishing perfusion at the site of ischemia through an infarct-directed intervention is a logical approach. Infarct-targeted intervention is the standard of care in ST elevation myocardial infarction (STEMI) and improves outcomes in ischemic stroke.^21,22^ Guidelines for the treatment of CLTI include identifying the ideal target arterial pathway (TAP) to restore in-line pulsatile flow to the ankle and foot; however, the TAP often involves the least diseased pathway and may not consider the location of the tissue loss.^23^

Primary procedural interventions for CLTI include the surgical and endovascular approaches. Both approaches have been determined to reduce AFS and mortality.^1,24^ Endovascular devices and open bypass graft materials have variable efficacy and durability depending on the device or material applied. Drug-coated technologies have shown large variability in effectiveness across the claudicate populations, with many trials reporting significant differences compared to POBA.^25–27^ Data regarding the drug coated balloon (DCB) technology and CLTI are limited; although freedom from major target limb amputation at 60 months has been reported as high as 90%.^28^ Likewise, advancing stenting technologies have also shown differences across the claudicate population, and while also not well studied in the CLTI population, AFS has been shown with interwoven nitinol stenting to be as high as 81% at 36 months.^29,30^ Similarly, open surgical revascularization outcomes may depend on the vein graft material used.^31^ The choice of the device or technique likely plays a significant role in the durability and success of any intervention.

Treatments and risk factors associated with wound healing are often not evaluated when analyzing patient outcomes. Off-loading, debridement, advanced wound care techniques, and the participation of a multidisciplinary team have all been shown to affect clinical outcomes.^32–34^ Wound healing is multifactorial and includes physiologic risk factors such as PAD, sensory neuropathy, glucose control, and renal disease, as well as treatment factors including off-loading, wound debridement, and advanced treatments. Intensive glycosylated hemoglobin control has demonstrated a 35% reduction of amputation risk.^35^ Off-loading techniques have shown wide efficacy in the treatment of wounds. Total contact casts are the gold standard to off-load the foot and have shown wound healing rates as high as 90% in 6 weeks.^36^ Patients that receive off-loading shoes following amputation have a 40% reduction of re-amputation compared to those that do not.^37^ Of the 9 studies evaluated, only 2 studies discussed the use of off-loading devices. In these two studies, there was no algorithm as to how a device was chosen, what devices were chosen, or patient compliance to the device. Advanced wound care therapies such as negative pressure wound therapy, split skin thickness grafting, and hyperbaric oxygen have also shown efficacy in improving CLTI wounds.^38–40^ Although numerous studies made note of such therapies, the number of patients that received these interventions was not quantified.

There were some inherent limitations in this review. As reported, there are 2 approaches to direct revascularization: surgical and endovascular. There is ongoing debate on whether one approach has improved outcomes over the other and may confound the outcomes that this review addresses. This limitation was addressed partially in a sub-analysis that examined only the studies that followed a surgical approach, which reported a similar finding as with all articles included. However, future review studies may benefit by encompassing a larger case load for both techniques as novel trials of angiosome continue to be produced. As mentioned earlier, there were also few specifications for the type of advanced wound care across multiple studies. Some articles mentioned techniques including off-loading, negative pressure wound therapy, and hyperbaric oxygen therapy; however, this review was unable to standardize the follow-up protocols. It would be interesting if future studies were able to incorporate these advanced techniques into the management for CLTI wounds to determine the effect they have on the healing process.

## Conclusion

Direct revascularization of the target angiosome to provide perfusion to the wound is logical and has shown improvement over indirect revascularization in this study. However, the importance of the intervention chosen, postintervention care, and optimization of physiological variables are important factors in the wound-healing paradigm.

## Supplemental Material

sj-docx-1-jet-10.1177_15266028241248524 – Supplemental material for Outcomes for Patients With Chronic Limb-Threatening Ischemia After Direct and Indirect Endovascular and Surgical Revascularization: A Meta-Analysis and Systematic Review Supplemental material, sj-docx-1-jet-10.1177_15266028241248524 for Outcomes for Patients With Chronic Limb-Threatening Ischemia After Direct and Indirect Endovascular and Surgical Revascularization: A Meta-Analysis and Systematic Review by Arthur Tarricone, Allen Gee, Karla de la Mata, Lee Rogers, Jose Wiley, Lawrence A. Lavery and Prakash Krishnan in Journal of Endovascular Therapy
