# Supplementary material for: Outcomes for Patients With Chronic Limb-Threatening Ischemia After Direct and Indirect Endovascular and Surgical Revascularization: A Meta-Analysis and Systematic Review
Source: J Endovasc Ther. 2024 Apr 30;33(1):56–63. doi: 10.1177/15266028241248524 (PMC12804421; doi:10.1177/15266028241248524)
Supplement: sj-docx-1-jet-10.1177_15266028241248524 – Supplemental material for Outcomes for Patients With Chronic Limb-Threatening Ischemia After Direct and Indirect Endovascular and Surgical Revascularization: A Meta-Analysis and Systematic Review [file sj-docx-1-jet-10.1177_15266028241248524.docx]

Search Strategy

Search: Angiosome And Revascularization

(("angiosomal"[All Fields] OR "angiosome"[All Fields] OR "angiosomes"[All Fields]) AND ("revascularisation"[All Fields] OR "revascularisations"[All Fields] OR "revascularise"[All Fields] OR "revascularised"[All Fields] OR "revascularising"[All Fields] OR "revascularization"[All Fields] OR "revascularizations"[All Fields] OR "revascularize"[All Fields] OR "revascularized"[All Fields] OR "revascularizes"[All Fields] OR "revascularizing"[All Fields]))

Results: 137

Final: 3

Search: Angiosome AND Direct Revascularization AND Indirect Revascularization

(("angiosomal"[All Fields] OR "angiosome"[All Fields] OR "angiosomes"[All Fields]) AND (("direct"[All Fields] OR "directed"[All Fields] OR "directing"[All Fields] OR "direction"[All Fields] OR "directional"[All Fields] OR "directions"[All Fields] OR "directivities"[All Fields] OR "directivity"[All Fields] OR "directs"[All Fields]) AND ("revascularisation"[All Fields] OR "revascularisations"[All Fields] OR "revascularise"[All Fields] OR "revascularised"[All Fields] OR "revascularising"[All Fields] OR "revascularization"[All Fields] OR "revascularizations"[All Fields] OR "revascularize"[All Fields] OR "revascularized"[All Fields] OR "revascularizes"[All Fields] OR "revascularizing"[All Fields])) AND (("indirect"[All Fields] OR "indirects"[All Fields]) AND ("revascularisation"[All Fields] OR "revascularisations"[All Fields] OR "revascularise"[All Fields] OR "revascularised"[All Fields] OR "revascularising"[All Fields] OR "revascularization"[All Fields] OR "revascularizations"[All Fields] OR "revascularize"[All Fields] OR "revascularized"[All Fields] OR "revascularizes"[All Fields] OR "revascularizing"[All Fields])))

Results: 54

Final 5

| Embase/Medline | Results |
| --- | --- |
| 1. Angiosome | 433 |
| 1. Direct Revascularization | 3503 |
| 1. Indirect Revascularization | 940 |
| 1. Chronic Limb Threatening Ischemia | 1309 |
| 1. 1 and 2 | 66 |
| 1. 1 and 3 and 4 | 9 |
| 1. 1 and 2 and 4 | 10 |

| Cochrane |
| --- |
| 1. Angiosome |
| 1. Direct Revascularization |
| 1. Indirect Revascularization |
| 1. Chronic Limb Threatening Ischemia |
| 1. 1 AND 2 AND 4 OR 1 AND 3 AND 4 |
| 1. Full Text |
| 1. English Only |
| 1. Comparative Trials, Randomized Controlled Trials, Case Controlled Studies, Prospective Studies, Retrospective Studies |

| Web Of Science |
| --- |
| 1. Angiosome |
| 1. Direct Revascularization |
| 1. Indirect Revascularization |
| 1. Chronic Limb Threatening Ischemia |
| 1. 1 AND 2 AND 4 OR 1 AND 3 AND 4 |
| 1. Full Text |
| 1. English Only |
| 1. Comparative Trials, Randomized Controlled Trials, Case Controlled Studies, Prospective Studies, Retrospective Studies |

Supplementary Figure 1

Supplementary Figure 2

Eggers Test showed no indication of publication bias (p=0.21)
